# Effect of a New Additive Based on Textile Fibres from End-of-Life Tyres (ELT) on the Mechanical Properties of Stone Mastic Asphalt

**DOI:** 10.3390/polym15071705

**Published:** 2023-03-29

**Authors:** Gonzalo Valdés-Vidal, Alejandra Calabi-Floody, Cristian Mignolet-Garrido, Cristian Díaz-Montecinos

**Affiliations:** 1 Department of Civil Engineering, Universidad de La Frontera, Temuco 4811230, Chile; 2 CDI—Centro de Desarrollo e Investigación, Maipú 9260061, Chile

**Keywords:** stone mastic asphalt (SMA), end-of-life tyres (ELT), ELT-based additive (WTTF), cellulose fibre, performance properties

## Abstract

Stone Mastic Asphalts (SMA) are asphalt mixes with discontinuous granulometry and a high content of asphalt binder. In order to prevent draindown of the asphalt binder and ensure good performance, these mixes must be strengthened with cellulose or mineral fibres and/or polymer additives. This study was designed to evaluate the effect of a granular additive based on waste tyre textile fibres (WTTF), developed as a replacement for cellulose commercial additives in SMA mixes. Use of the WTTF-based additive will encourage the development of sustainable mixes by recycling a by-product of end-of-life tyres (ELT), which currently constitute a major environmental problem around the world. To this end, in the present experimental study we evaluated the replacement of cellulose-based commercial fibre with different percentages of WTTF-based additive (0%, 50%, 75%, 100%) in an SMA asphalt mix. The following design and performance properties were evaluated: resistance to cracking, stiffness modulus, sensitivity to moisture, and resistance to permanent deformation. The results indicated that replacing 100% of the cellulose commercial additive in the SMA mix by the WTTF-based additive allowed the mix to meet its design properties and showed good performance in the mechanical properties evaluated, with behaviour similar to that of the reference SMA mix.

## 1. Introduction

Asphalt paving is the most widely used road-surfacing material in the world, used on around 95% of paved roads [1]. This is largely because hot asphalt mixes (HMA) offer very good mechanical performance in paving. These mixes consist of approximately 5% asphalt binder [2], which acts as an impermeable, cohesive matrix, binding the stone aggregates together and ensuring the durability of the asphalt mix [2,3]. However, this organic material is highly susceptible to thermal conditions [4], being solid at low temperatures, visco-elastic at intermediate temperatures and viscous at high temperatures [5]. This, combined with the velocity and volume of the traffic load on road paving, has a direct impact on the behaviour of HMA mixes, leading to failure by thermal cracking [6], fatigue cracking [7,8] and permanent deformation [9], among other kinds of deterioration. In this context, when traffic and/or environmental conditions are particularly demanding, it becomes necessary to use asphalt mixes with better structural and functional performance in order to satisfy the demands of paving use. The physical and volumetric characteristics of Stone Mastic Asphalt (SMA) give it a better mechanical performance than conventional asphalt mixes, especially in terms of resistance to the forces which lead to deteriorations such as permanent deformation and fatigue [10]. SMA mix is a type of hot asphalt mix with discontinuous granulometry, characterised by a high content of asphalt binder (6.0–7.0%), either conventional or modified with polymer [11]. It also has a strong mineral skeleton of coarse aggregates (70–80% weight) [11,12,13], the particle gradation of which gives the paving better structural and functional performance in terms of the main types of deterioration to which it is exposed. Due to their high asphalt binder content, SMAs require the addition of stabilising additives to avoid draindown of the asphalt binder in the mix [14,15]. Stabilising additives commonly used in SMA are based on cellulose or mineral fibres and polymeric additives [16]. Cellulose fibre, polyester fibre, lignin fibre and glass fibre, among others, have an effect on the performance of mixes, improving their dynamic module properties, their resistance to permanent deformation, sensitivity to moisture, and resistance to fatigue damage; they also reduce draindown of the asphalt binder [13,15,17]. Furthermore, when these asphalt mixes are prepared with asphalt binders modified with SBS (styrene–butadiene–styrene, a polymer additive commonly used in the modification of asphalt binders), the combined effect with the use of fibre allows a substantial improvement in the mechanical performance of the SMA at high, intermediate and low service temperatures [15,16].

In recent years, the growth of the automobile industry has led to an increase in the numbers of end-of-life tyres (ELT), which have now become a major concern all over the world due to the environmental, health and social problems that they represent [18,19,20]. It is estimated that more than 20 million metric tons are accumulated worldwide every year, of which around 70% are recovered by re-use and recycling of the ELT [21]; the remainder are deposited in landfill or dumped illegally. The EU is the main player in recycling, recovering approximately 90% of the ELT generated; it is followed by USA (81%) and China (60%) [22,23]. In Latin America, the pertinent regulations and ELT recovery processes are quite precarious compared to the EU and USA. ELT are used principally as an additional fuel in cement plants [23], but this practice is increasingly restricted by environmental regulations [22]. The principal products of ELT recycling are rubber (natural or synthetic), which accounts for 45–47% of the weight; steel fibres, 12–24% of the weight; textile fibres, 1–10% of the weight, and other components equivalent to 29–34% of the weight [24,25,26]. Rubber is widely used in the recycling industry, being incorporated as an alternative polymer to improve the performance of hot asphalt mixes and cement; it is also used as a filler in infrastructure works, sports surfaces, athletics tracks, and acoustic and thermal insulation [27,28,29,30,31,32,33]. The steel fibres recovered are used as raw material for producing virgin steel and as an additive in cement [34]. There is currently no industrial use for the textile fibre from ELT, which ends up in landfill or incinerators [34,35]; a small amount is used as combustion material in cement plants [36]. The textile fibre obtained from ELT recycling generally consists of polyester and polyamide (nylon 6 or nylon 6.6) [26,34,35]. Studies exist in which polyester fibres and nylon fibres have been used in SMA mixes. Sheng et al. (2017) [37] studied the impact of polyester and mineral fibres in the design of SMA mixes, finding that these fibres give improved performance in the Marshall stability, air void content and voids in mineral aggregate (VMA) compared with the reference SMA mix of the study. Yin and Wu (2018) [13] evaluated the effects of the addition of nylon to SMA mixes, finding that it improved the stability of the mix at high temperature, its resistance to cracking at low temperature and its sensitivity to moisture. Putman and Amirkhanian (2004) [38] studied the viability of using ELT fibres in SMA mixes, compared with cellulose and polyester fibres. They found that ELT fibres were a promising alternative as a stabilising additive for SMA, without affecting the mechanical properties and binder draindown of the reference mix. Calabi et al. (2022) [39] showed that the use of ELT as an additive for asphalt binders improved performance at high temperatures, which was subsequently analysed in asphalt mixtures produced using asphalt binders with the addition of ELT, obtaining similar results (Calabi et al. 2022) [40]. Valdés et al. (2022) developed an additive based on ELT fibre (WTTF) for incorporation into HMA mixes, finding a significant improvement in the stiffness modulus and the resistance to permanent deformation and moisture damage [26]. 

Based on the above study by Valdés et al. (2022) [26], in the present investigation a sustainable additive was developed for use in SMA mixes as a replacement for the cellulose fibres usually used as a stabilising additive. The sustainability of this new additive is attributable mainly to the fact that it is manufactured from WTTF, a mass waste product [41]. In view of the benefits of the incorporation of nylon and polyester fibres in SMA mixes recorded in the literature [13,37,38], the new WTTF-based additive for SMA mixes shows great promise as an absorbent stabiliser, preventing draindown of the asphalt binder without affecting the design properties and performance of the mix. The aim of this experimental study was, therefore, to evaluate the effect of this new additive on the mechanical design properties and performance of the SMA, comparing it with a reference SMA mix using a cellulose-based commercial stabilising additive. Below, we describe the composition of the new additive developed for the experiment and the reference SMA mix. For the experimental design, we determined three percentage contents of the cellulose commercial additive which would be replaced by the new WTTF-based additive. We then evaluated the mechanical properties related to the performance of the asphalt mixes: resistance to cracking, stiffness modulus, sensitivity to moisture and resistance to permanent deformation.

## 2. Materials and Methods

### 2.1. WTTF-Based Additive

**Figure 1:**
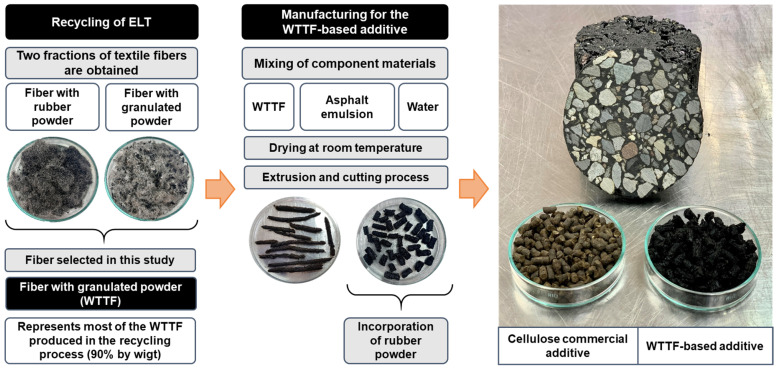
Development of the WTTF-based additive.

For the present study, an additive was developed from waste tyre textile fibre (WTTF) to replace the cellulose commercial additive usually used in the manufacture of SMA asphalt mixes. The WTTF-based additive for SMA mixes developed for this study adopted the same dosage of materials used in a previous investigation, in which an additive was developed to improve the performance of conventional HMA mixes [26]. The principal components of the WTTF-based additive are carbon and oxygen, with small fractions of sulphur, silicon, iron and sodium [26]. This is the normal elemental composition of WTTF, in which the main polymer is polyester [26], as previously reported by Bocci and Prosperi (2020) [34]. The materials used were: WTTF, rapid-setting cationic asphalt emulsion, and water (evaporated during setting of the emulsion) in a proportion of 1:1:1 (weight). The additive was obtained in granular format by extrusion and cutting; rubber powder was added in a proportion of 1:20 to prevent agglomeration of the additive. Figure 1 shows the development of the granular WTTF-based additive; its characterisation is given in Table 1.

**Figure 2:**
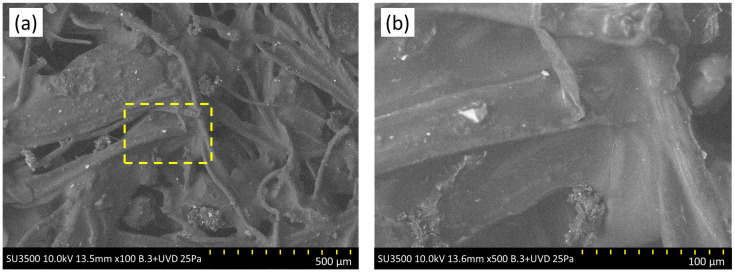
SEM images of the WTTF-based additive used in this study: (**a**) 500 μm; (**b**) 100 μm.

**Figure 3:**
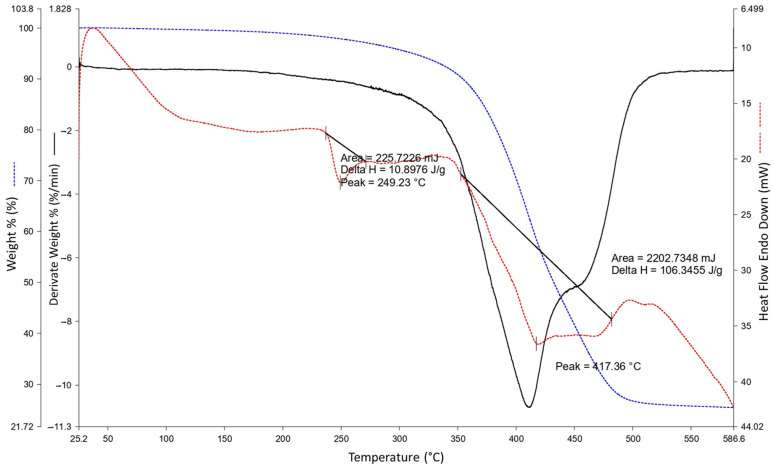
Temperature TGA profile of the WTTF-based additive used in this study.

The morphological characteristics of the WTTF-based additive were studied by scanning electron microscope (SEM) at different magnifications (Figure 2). As shown in Figure 2a, the WTTF filaments are completely coated with asphalt cement and rubber particles, forming a sort of three-dimensional network, generating jointly a rough, compact structure. In Figure 2b, small, light-coloured particles can be observed adhering to the WTTF. These could be fragments of steel fibres derived from tyre recycling [23]. Furthermore, thermogravimetric analysis (TGA) was used to determine the state of decomposition of the WTTF-based additive. Figure 3 indicates that the first phase change or melting point occurs around 242 °C (with peak at 249.23 °C), which coincides with the melting point of polyester [42]. Subsequently, the temperature profile shows an increase in mass loss, characteristic of the change from the liquid phase to the gas phase. This indicates that the decomposition point of the WTTF-based additive occurs at 417.36 °C; this coincides with the decomposition point of polyester, which occurs at above 365 °C [43].

### 2.2. Cellulose Commercial Additive

SMA mixes have a high content of asphalt binder; additives must, therefore, be used to stabilise the mix, preventing asphalt draindown. In this study, the reference mix contained a cellulose commercial additive, the main characteristics of which are shown in Table 2.

**Figure 4:**
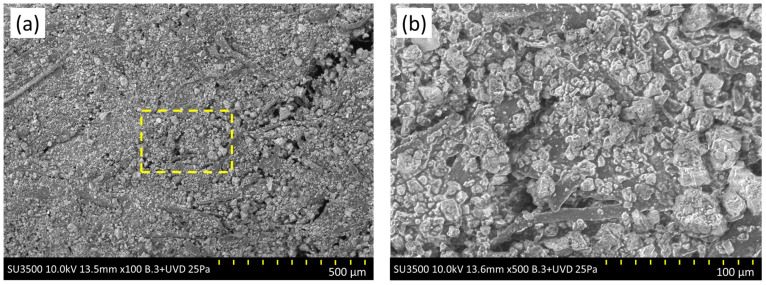
SEM images of the cellulose commercial additive used in this study: (**a**) 500 μm; (**b**) 100 μm.

**Figure 5:**
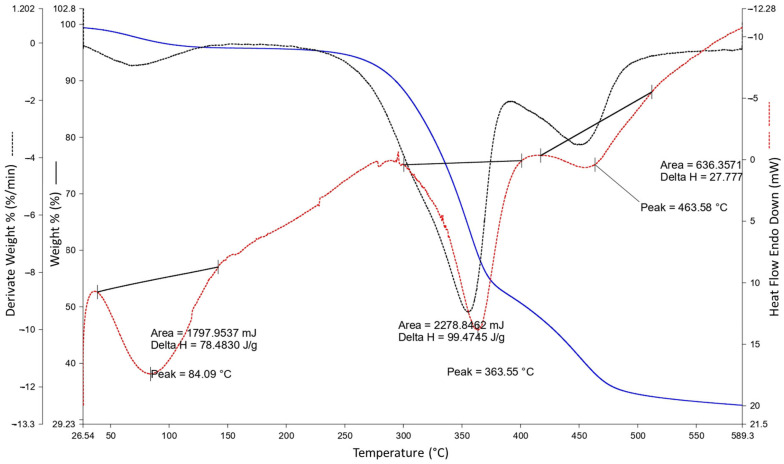
Temperature TGA profile of the cellulose commercial additive used in this study.

The morphological characteristics of the cellulose commercial additive were studied by scanning electron microscope (SEM) at different magnifications (Figure 4). Figure 4a shows a rough, homogeneous surface, with the presence of smooth filaments of cellulose fibre. In Figure 4b, the rough surface is observed to consist of a light-coloured, straight-edged structure, indicating the presence of an inorganic substance. This could indicate the presence of calcium carbonate, an additive commonly used as a covering agent in the manufacture of paints and plastics [45]. Beneath the inorganic structure, a dark surface can be observed which would be the asphalt cement used to bind the cellulose fibres. Thermogravimetric analysis (TGA) was used to determine the state of decomposition of the cellulose commercial additive. Figure 5 shows that, in the first section, a mass loss occurs below 100 °C, indicating the loss of the moisture contained in the sample analysed. Then, at around 260 °C, the first phase change or melting point occurs, leading on to the decomposition phase at 363.55 °C; this agrees with the decomposition point of the cellulose, which occurs in the range 350–400 °C [46]. The asphalt cement contained in the cellulose commercial additive presents different decomposition phases, indicating a peak at 463.58 °C. The SEM images indicate the presence of crystals of calcium carbonate; decomposition of this substance was not reported by the TGA, as it occurs at above 600 °C [47].

### 2.3. Asphalt Binder

The asphalt binder used in this study was an asphalt cement modified with polymers, penetration 60/80, complying with Chilean standards. The properties of the asphalt binder are shown in Table 3.

### 2.4. Aggregates

The aggregates used were of riverbed origin and complied with the Chilean standard for use in surface courses. The aggregates contained, principally, particles of dolomite, basalt, dacites, andesites, rhyolites, sandstone, quartz and quartzite. By weight, 8% of the mineral filler (lime) was added to the aggregates. The properties of the aggregates used are shown in Table 4.

**Figure 6:**
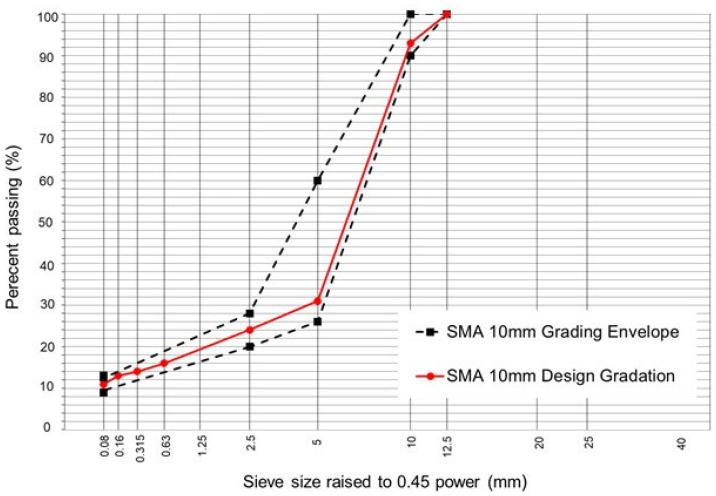
Design gradation of the SMA10 asphalt mixture.

The fractions of aggregates, including the mineral filler, were combined with a maximum nominal granulometric band of 10 mm (SMA10), as per Chilean standards [50]. The gradation of the aggregates is given in Figure 6.

## 3. Experimental Design

**Figure 7:**
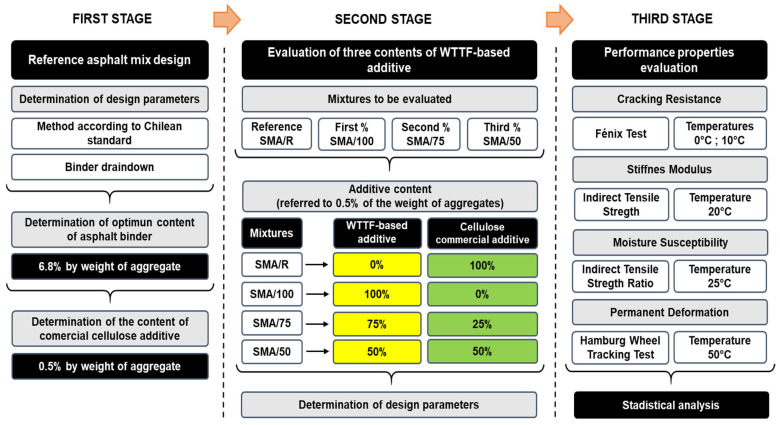
Experimental stages used in the study.

This study evaluated the effect of the granular WTTF-based additive on the design and performance properties of an SMA mix [50]. Figure 7 shows the experimental design of the study. In the first stage, the reference mix (SMA/R) was designed with the characteristics usually used in surface courses. In the second stage, we evaluated the effect on the design properties of the SMA mix of adding three amounts of granular WTTF-based additive in the place of cellulose commercial additive. The replacement proportions used were 0%, 50%, 75% and 100% of the content of cellulose commercial additive in the SMA mix. In the third stage, we evaluated the performance properties of the asphalt mixes studied, checking the significance of the results by statistical analysis. 

### 3.1. Mix Design

The SMA/R mix was designed by the method established in the Chilean standards for the manufacture of SMA mixes [50]. The optimum content of asphalt, determined to ensure 4% air voids in the mix, was 6.8% by weight of the aggregates (6.5% by weight of the mix), using 0.5% of cellulose commercial fibre by weight of aggregates. The design parameters of the SMA/R mix were checked, to ensure that it complied with the minimum content of cellulose fibres, minimum content of asphalt, air voids in the mix, voids in the mineral aggregate (VMA), voids in the coarse aggregates (VCA) and binder draindown at the mixing temperature. 

Different contents of WTTF-based additive were selected in this study to evaluate the behaviour of the SMA mixes. SMA mixes were manufactured with 0%, 50%, 75% and 100% replacement of the cellulose commercial additive content used in the SMA/R mix (0.5% by weight of aggregates). All the asphalt mixes evaluated were manufactured with an optimum asphalt content of 6.8% (by weight of aggregates), at manufacture and compaction temperatures of 177 ± 5 °C and 165 ± 5 °C, respectively. The design properties of the SMA mixes manufactured with the WTTF-based additive were checked to ensure that they complied with the specifications of Chilean standards, as shown in Table 5.

The evaluations of binder draindown [51] show that all the mixes evaluated presented draindown of less than 0.3%, as specified for this type of mix. Therefore, the WTTF-based additive prevents the asphalt binder from draining through the aggregate, with a draindown value similar to that of the mix with cellulose commercial additive. 

### 3.2. Testing Methods

To determine the effect of the use of the WTTF-based additive in the SMA mixes, the mechanical performance properties of the mixes in paving were evaluated. In this study we evaluated the resistance to cracking at low temperature, the stiffness modulus, the sensitivity to moisture damage and the resistance to permanent deformation.

The resistance to cracking at low temperature of the mixes evaluated was determined by the Fénix test, which simulates Mode I fractures, the principal cracking mechanism [52].

**Figure 8:**
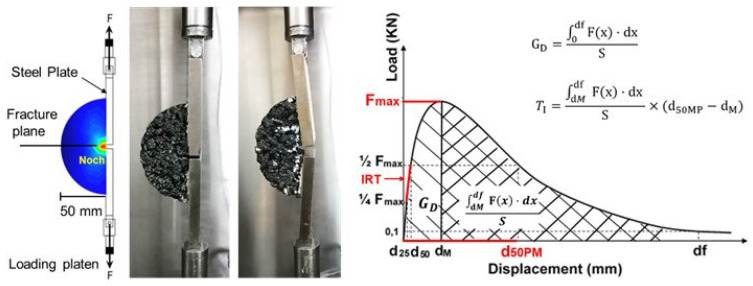
Fénix test setup and load-displacement output test curve.

The Fénix test consists of applying a tensile force to a crack induced in a cylindrical test specimen cut in half; the crack is propagated by the application of force, as shown in Figure 8. Three test specimens were made for each type of mix evaluated. In each semi-circular test specimen, a 5 mm crack was induced across the diametral face. Two steel plates were fixed to the flat face of the section, either side of the notch; they were connected to the press by a ball-joint, allowing movement of the fixture points. A tensile force was applied at a constant velocity of 1 mm/min, until the material fractured. The parameters evaluated to describe the behaviour of the mixes were: maximum tensile force (Fmax), tensile stiffness index (TSI), displacement at 50% of post-maximum tensile force (d50PM), dissipated energy per unit area (GD), and toughness index (TI). The temperatures evaluated were 0 °C and 10 °C.

The stiffness modulus (*S_M_*) was determined by the indirect tensile strength test following European Standard UNE-EN 12697-26. The test consists of applying a pulsed load to one of the diametral planes of a cylindrical test specimen, recording the variation in the load applied and the horizontal diametral deformation over time, and also determining the area of load. Three cylindrical test specimens were produced for each type of mix evaluated; they were kept at a controlled temperature of 20 °C for 24 h before testing. The stiffness modulus (*S_M_*) was calculated by Equation (1).

(1) $$S_{M}=\frac{F\cdot \left(v+0.27\right)}{\left(z\cdot h\right)}$$

where *S_M_* is the stiffness modulus measured in (MPa), *F* is the maximum vertical load applied in (N), *v* is Poisson’s ratio, *z* is the horizontal displacement in (mm), and *h* is the average thickness of the specimen in (mm).

The sensitivity to moisture was evaluated by the indirect tensile strength ratio (*ITSR*) as per European Standard UNE-EN 12697-12. This method uses the indirect tensile strength (ITS) parameter of dry and wet test specimens under European Standard UNE-EN 12697-23 to evaluate the progressive degradation of the mechanical properties of the asphalt mixes under the action of water. Six cylindrical test specimens were made for each type of mix evaluated, divided into two subsets of three test specimens each, of identical physical characteristics. One of these subsets was conditioned at ambient temperature (20 ± 5 °C) for 72 h to determine the dry ITS. The other was used to determine the wet ITS; the test specimens in this subset were first saturated in distilled water at 20 ± 5 °C, under a vacuum pressure of 6.7 ± 0.3 kPa for 30 min, and then conditioned in a water bath for 72 h at 40 °C. Both sets of test specimens were then conditioned at the test temperature of 25 °C for 2 h. On completion of this period, the indirect tensile strength test was carried out; a force was applied at a displacement velocity of 50 mm/min, to failure of the material. The indirect tensile strength ratio (*ITSR*) was obtained by Equations (2) and (3).

(2) $$ITS=\frac{2\cdot P}{\pi \cdot D\cdot H}\cdot 100$$

(3) $$ITSR=\frac{ITS_{w}}{ITS_{d}}\cdot 100$$

where *ITS* is the indirect tensile strength (kPa), *P* is the maximum applied load (kN), *D* is the specimen diameter (mm), *H* is the specimen height (mm), *ITS_w_* is the mean resistance to indirect tensile strength of the wet-conditioned test samples (Pa) and *ITS_d_* is the mean resistance to indirect tensile strength of the dry-conditioned test samples (Pa).

The resistance of the mixes to permanent deformation was evaluated by the Hamburg Wheel Tracking Test (HWTT), following AASHTO Standard T324. This test determines the degree of permanent deformation and stripping in asphalt mixes due to a loss of adhesion between the asphalt binder and the aggregates. It consists of subjecting cylindrical test specimens submerged in a water bath at 50 ± 0.5 °C to a constant load of 705 ± 4.5 (N) for 10,000 cycles (or 20,000 passes). Four cylindrical test specimens were made for each type of mix evaluated in the trial. The parameters evaluated were the mean rut depth (RD) and the slope of the deformation curve between cycle 5000 and cycle 10,000 (WTS).

The significance of the results ob”aine’ In the trials of the mechanical properties of the asphalt mixes was verified by statistical analysis with a confidence level of 95%. The statistical tests used were determined by the verification of the assumptions of normality and homoscedasticity of the data, using the Shapiro–Wilk test and Levene’s test, respectively. The data with normal distribution were analysed by analysis of variance (ANOVA). The Kruskal–Wallis H test was used for data which did not have a normal distribution, in which case the samples tested were considered to be independent.

## 4. Analysis of Results

**Figure 9:**
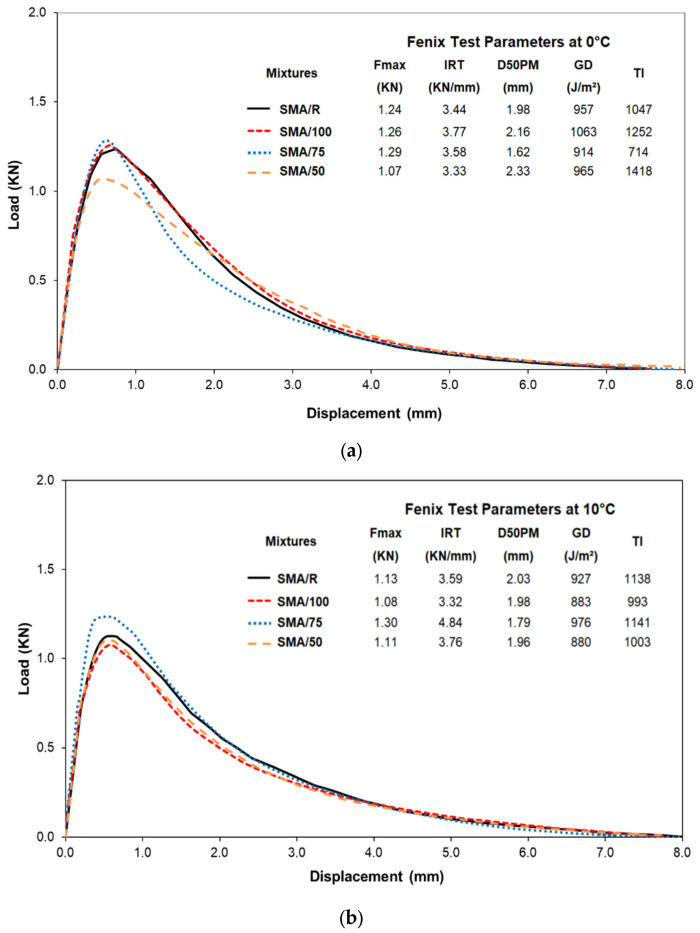
Fénix test parameters at different temperatures: (**a**) 0 °C; (**b**) 10 °C.

The effect of the WTTF-based additive on resistance to cracking at low temperature was determined by evaluation of the main parameters given by the Fénix test, as shown in Figure 9. The load-displacement curve for the behaviour of the mixes at 0 °C is shown Figure 9a. This graph shows very similar behaviour between the reference SMA/R mix and the mix using a 100% replacement of the commercial additive with the new WTTF-based additive, SMA/100. The maximum tensile force (Fmax) and the tensile strength index (TSI) show that the SMA/100 mix has the same resistance to tensile force, as it has a similar TSI value to that of the SMA/R. The measurement of the flexibility prior to breaking (d50PM) shows that the SMA/100 mix has a value 9% higher than that of the SMA/R reference mix. Hence, we conclude that replacing 100% of the cellulose commercial additive with the WTTF-based additive produces mixes with a similar tensile strength, without affecting their resistance to deformation at lower temperatures. These results agree with the results obtained for the energy dissipated in cracking (GD) and the toughness index (TI), which show that cracking demanded more energy in the SMA/100 mix than in the SMA/R reference, just as more energy was demanded to cause cracking in the post-maximum tensile force zone. However, the differences between the two mixes in these parameters were small. No clear trend could be identified in the parameters evaluated for the SMA/75 and SMA/50 mixes at 0 °C. The results obtained at 10 °C are shown in Figure 9b. At 10 °C, we see that, in general, all the mixes using the WTTF-based additive presented similar behaviour to the SMA/R in the mechanical parameters involved in cracking of asphalt mixes, with the exception of the test with 75% replacement of the cellulose commercial additive, SMA/75, which produced higher resistance to maximum force (Fmax) and stiffness (TSI). Based on these results we observe that at both 0 °C and 10 °C the SMA/100 mix presented good performance in the parameters evaluated, with behaviour very similar to that of the reference mix. 

The analysis of variance (ANOVA) applied to the results obtained in the Fénix parameters for each test temperature indicate that the content of WTTF-based additive presented no significant differences from the SMA/R reference mix in the performance parameters evaluated, with a *p*-value above the level of significance (0.05) at 95% confidence. This shows that the same performance can be obtained in this property when the cellulose commercial additive is replaced with the WTTF-based additive, as in the SMA/R.

**Figure 10:**
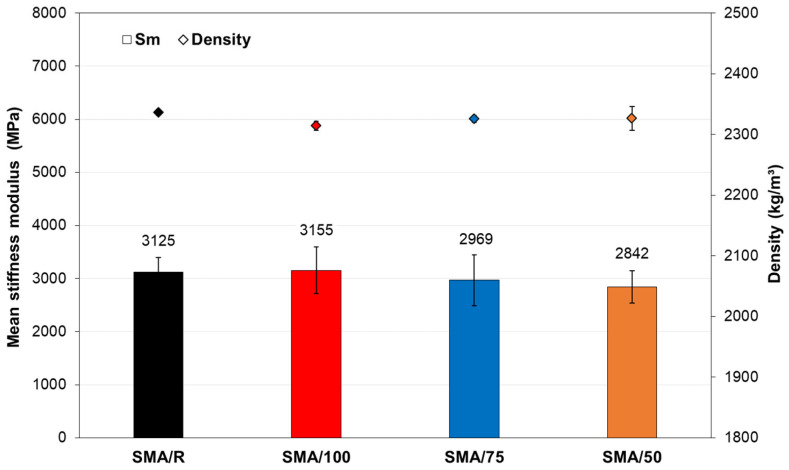
Stiffness modulus of the SMA mixtures studied at 20 °C.

The results obtained for the stiffness modulus at 20 °C are shown in Figure 10. The graph shows that the results are very similar for all the mixes tested. This agrees with the statistical results of the Kruskal–Wallis H Test, which indicate that the mixes with WTTF-based additives presented no significant differences (*p*-value = 0.46 > 0.05) from the reference mix. This shows that the use of WTTF-based additive to replace the cellulose commercial additive used in SMA mixes did not affect the mechanical response of the stiffness modulus, and that the structural performance of the SMA/R was maintained in the mixes with different additive replacement percentages. This result agrees with Mokhtari and Moghadas (2012) [14], who found that a similar effect can be obtained in the stiffness behaviour of the SMA mix by the addition of mineral fibre as by the addition of cellulose fibre, just as we observed with the WTTF-based additive. Other studies have shown that the use of fibres of mineral and synthetic origin in SMA mixes strengthens the aggregate–binder matrix of the mix, producing good performance in the stiffness modulus [13,53]. This is important, as the principal component of the WTTF-based additive is a synthetic fibre [34], which could explain the good performance of the SMA/100 mix for this property. The mixes with WTTF-based additive presented similar density values to those of the SMA/R. 

**Figure 11:**
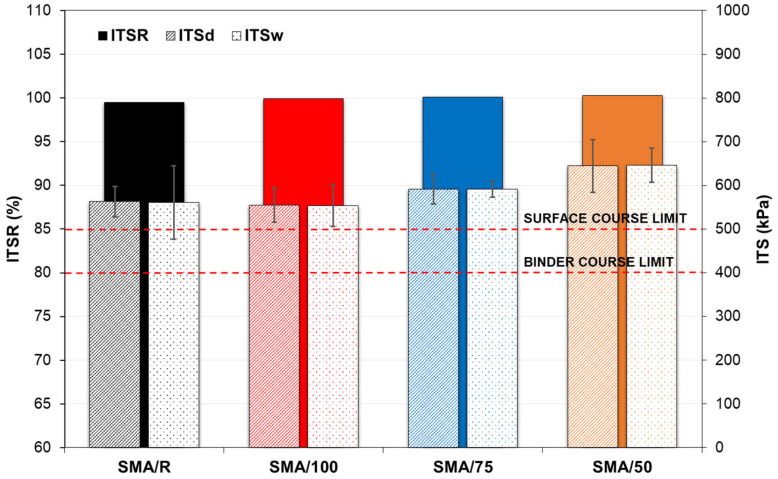
Moisture sensitivity test results.

The indirect tensile strengths of the SMA mixes evaluated in dry $\left(ITS_{d}\right)$ and wet conditions $\left(ITS_{w}\right)$ are shown in Figure 11. The results obtained show that the values recorded for the SMA mixes with WTTF-based additives were similar to those obtained for the reference mix. This agrees with the statistical results of the Kruskal–Wallis H Test, which indicate that the mixes tested presented no significant differences in $ITS_{d}$ (*p*-value = 0.078 > 0.05) and $ITS_{w}$ (*p*-value = 0.108 > 0.05). The good performance for this property observed in the SMA mixes with the WTTF-based additive may be related to the characteristics of the fibre used in the additive, which strengthened the internal matrix of the SMA mix, preventing its internal structure from being affected by the action of water, as occurred in another study in which a nylon-based synthetic fibre was used as the additive [13]. All the mixes tested showed a similar performance for sensitivity to moisture, complying with the minimum values required by European standards for base and intermediate courses (*ITSR* > 80%) and surface courses (*ITSR* > 85%) [54]. This effect agrees with the results obtained by Herráiz et al. (2016) [55], who evaluated the incorporation of different fibres in SMA mixes, finding that the use of polyester fibre has a positive effect against the action of water, indicating a higher variation in the indirect tensile strength ratio (*ITSR*) that that of the reference mix with cellulose fibre. Thus, the use of synthetic fibres has a positive effect on the behaviour of SMA mixes in terms of sensitivity to moisture. Based on the results obtained, it can be said that the use of the WTTF-based additive does not affect the internal adhesion and cohesion capacities in the aggregate–binder matrix of the SMA mix, demonstrating the potential of the WTTF-based additive to resist moisture damage.

**Figure 12:**
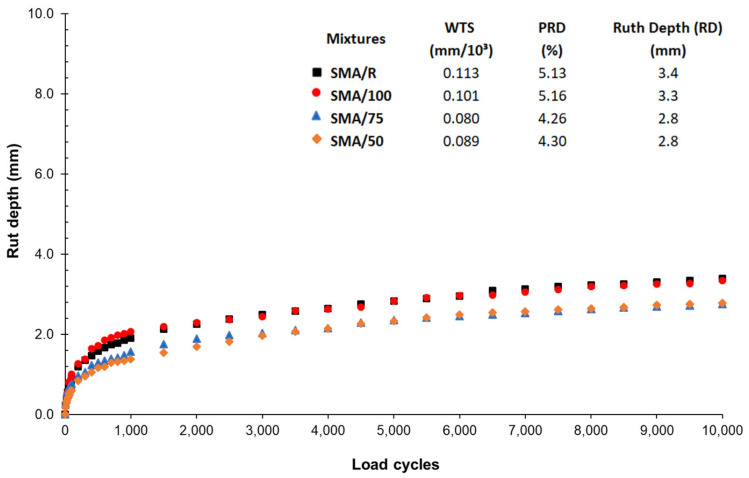
Hamburg wheel tracking test results at 50 °C.

The results of the Hamburg Wheel Tracking Test for the rutting depths (RD) recorded for the SMA mixes after different load cycles are shown in Figure 12. All the SMA mixes tested with the WTTF-based additive presented a low deformation rate, with a maximum difference in RD from that of the SMA/R at cycle 10,000, of 0.6 mm. The RD parameter shows that the use of the WTTF-based additive had no significant effect on the performance of the SMA mixes tested; the Kruskal–Wallis H Test gave a *p*-value = 0.621, above the level of significance (0.05). This effect may be due to the fact that the WTTF and the rubber provided good cohesion and adhesion in the internal structure of the mix, thus preventing any reduction in their resistance to permanent deformation [15,38]. The use of synthetic fibres may contribute to the formation of asphalt mixes, which have better consistency at high temperatures due to the formation of a three-dimensional network in the matrix of the mix [13,56,57,58,59]. This agrees with the good behaviour of the WTTF-based additive in the resistance to permanent deformation of the SMA mix. 

The SMA mixes with WTTF-based additive present a slight fall in the deformation curve (WTS) between cycles 5000 and 10,000 compared to the SMA/R mix, indicating a slower evolution of damage. The results showed no change in the slope of the deformation curves, indicating that all the mixes tested presented a good performance for stripping, which is related to the loss of adhesion between the stone aggregates and the asphalt binder.

## 5. Conclusions

Cellulose-based additives in SMA mixes can be successfully replaced, in various percentages, by a new additive developed using WTTF, contributing to the circular economy of end-of-life tyres (ELT).

The volumetric and mechanical design properties of all the SMA mixes with the WTTF-based additive meet the design parameters established for surface courses. All the mixes presented less binder draindown than the maximum allowed, providing good performance in this parameter.

The evaluation of the resistance to cracking showed that the SMA mixes with WTTF-based additive at 0 °C and 10 °C produced a similar behaviour to that of the reference SMA/R mix in the different parameters assessed. However, at 0 °C the SMA/100 mix presented greater flexibility (d50PM), offering a more ductile and deformable response. This mix also required a high load to produce cracking (GD), and showed greater toughness (TI).

With respect to the stiffness at 20 °C, the use of a WTTF-based additive produced a stiffness modulus of the same order as the SMA/R mix, with no significant differences regardless of the percentage replacement used.

The sensitivity to moisture assessment showed that the SMA mixes with the WTTF-based additive presented no significant difference in the indirect tensile strength (dry and wet) compared to the reference mix SMA/R. Furthermore, all the mixes evaluated complied with the indirect tensile strength ratio required by European regulations for use in intermediate and surface courses.

SMA mixes with the WTTF-based additive presented a good performance in resistance to permanent deformation, with a rutting depth similar to that of the reference mix SMA/R. Stripping was not recorded for any of the mixes.

From the results obtained in this study, we may conclude that up to 100% of the cellulose commercial additive can be replaced with a WTTF-based additive in SMA mixes without affecting the design properties and performance of the material.

## Figures and Tables

**Table 1:** Characterization of the WTTF-based additive.

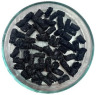	Diameter range (mm)	:	3.6–5.8
	Length range (mm)	:	4.8–12.1
	Apparent density (g/cm^3^)	:	0.25
	Real density (g/cm^3^)		1.18
	Melting point (°C)	:	242
	Weight composition [26]	:	WTTF (58%)
		:	Asphalt emulsion (37%)
		:	Rubber powder (5%)
	Asphalt emulsion used [26]	:	Cationic rapid-setting asphalt emulsion containing >65% residue asphalt

**Table 2:** Characterization of the cellulose commercial additive.

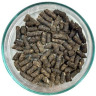	Diameter range (mm)	:	4.0–4.8
	Length range (mm)	:	4.3–12.4
	Apparent density (g/cm^3^)	:	0.38
	Real density (g/cm^3^)	:	1.49
	Melting point (°C)	:	260
	Weight composition [44]	:	Cellulose fibre (66%)
		:	Asphalt (34%)
	Asphalt used [44]	:	50/70 (0.1 mm)

**Table 3:** Properties of the asphalt binder used.

Tests	CA 60/80	Specs. [48]
Penetration at 25 °C, 100 g. 5 s. (0.1 mm)	62	60–80
Softening point R&B (°C)	72.4	Min. 60
Ductility at 25 °C, 5 cm/min, (cm)	112	Min. 80
Linear elastic recovery at 13 °C, 20 cm, 1 h, (%)	85	Min. 50
Elastic recovery by torsion at 25 °C, (%)	72	Min. 60
Penetration index	3.8	Min. +2.0
FRAASS breaking point, (°C)	−15	Max. −15
Flash point, (°C)	>300	Min. 235
Storage stability	<4	To be reported
Performance grade PG	64V(22)-28	To be reported

**Table 4:** Physical properties of aggregates used.

Tests	Results	Specs. [49]
***Coarse aggregate***		
Crushed aggregates (%)	96	Min. 90
Flakiness index (%)	13	Max. 25
Los Angeles abrasion loss (%)	14	Max. 25
Sodium sulphate soundness (%)	0.3	Max. 12
Static method adhesion	≥95	Min. 95
Dynamic method adhesion	≥95	Min. 95
***Fine aggregate***		
Plasticity index	Non-plastic	Non-plastic
Riedel-Weber adhesion	4–9	Min. 0–5
Soundness sodium sulphate (%)	1.0	Max. 15
***Combined aggregate***		
Soluble salts (%)	0	Max. 2
Sand equivalent (%)	53	Min. 50
Water absorption (%)	1.2	Max. 2

**Table 5:** Design parameters of the mixes evaluated.

Mix Type	WTTF-Based Additive	Cellulose Commercial Additive	Optimum Asphalt Content	Density	Air Voids	VMA	$VCA_{MIX}$	$VCA_{DRC}$	Binder Draindown
	(% Referred to 0.5% of Aggregate)		(% by Weight of Aggregate)	(kg/m^3^)	(%)	(%)	(%)	(%)	(%)
SMA/R	0	100	6.8	2.333	4.2	18.2	31.3	40.1	0.12
SMA/100	100	0	6.8	2.328	4.2	18.4	31.2	40.1	0.17
SMA/75	75	25	6.8	2.324	4.3	18.5	31.2	40.1	0.15
SMA/50	50	50	6.8	2.335	4.0	18.1	31.2	40.1	0.11
Chilean Specifications for surface course [50]					4	>17	${\mathrm{VCA}}_{\mathrm{MIX}}$ < ${\mathrm{VCA}}_{\mathrm{DRC}}$		Max 0.3

## Data Availability

Not applicable.
